# Monte Carlo Dosimetry of the ^60^Co BEBIG High Dose Rate for Brachytherapy

**DOI:** 10.1371/journal.pone.0139032

**Published:** 2015-09-29

**Authors:** Luciana Tourinho Campos, Carlos Eduardo Veloso de Almeida

**Affiliations:** Laboratório de Ciências Radiológicas (LCR/DBB/ UERJ), Rua São Francisco Xavier, 524 Maracanã, CEP: 205550, Rio de Janeiro, Brasil; North Shore Long Island Jewish Health System, UNITED STATES

## Abstract

**Introduction:**

The use of high-dose-rate brachytherapy is currently a widespread practice worldwide. The most common isotope source is ^192^Ir, but ^60^Co is also becoming available for HDR. One of main advantages of ^60^Co compared to ^192^Ir is the economic and practical benefit because of its longer half-live, which is 5.27 years. Recently, Eckert & Ziegler BEBIG, Germany, introduced a new afterloading brachytherapy machine (MultiSource^®^); it has the option to use either the ^60^Co or ^192^Ir HDR source. The source for the Monte Carlo calculations is the new ^60^Co source (model Co0.A86), which is referred to as the new BEBIG ^60^Co HDR source and is a modified version of the ^60^Co source (model GK60M21), which is also from BEBIG.

**Objective and Methods:**

The purpose of this work is to obtain the dosimetry parameters in accordance with the AAPM TG-43U1 formalism with Monte Carlo calculations regarding the BEBIG ^60^Co high-dose-rate brachytherapy to investigate the required treatment-planning parameters. The geometric design and material details of the source was provided by the manufacturer and was used to define the Monte Carlo geometry. To validate the source geometry, a few dosimetry parameters had to be calculated according to the AAPM TG-43U1 formalism. The dosimetry studies included the calculation of the air kerma strength *S*_*k*_, collision kerma in water along the transverse axis with an unbounded phantom, dose rate constant and radial dose function. The Monte Carlo code system that was used was *EGSnrc* with a new cavity code, which is a part of EGS++ that allows calculating the radial dose function around the source. The spectrum to simulate ^60^Co was composed of two photon energies, 1.17 and 1.33 MeV. Only the gamma part of the spectrum was used; the contribution of the electrons to the dose is negligible because of the full absorption by the stainless-steel wall around the metallic ^60^Co. The XCOM photon cross-section library was used in subsequent simulations, and the photoelectric effect, pair production, Rayleigh scattering and bound Compton scattering were included in the simulation. Variance reduction techniques were used to speed up the calculation and to considerably reduce the computer time. The cut-off energy was 10 keV for electrons and photons. To obtain the dose rate distributions of the source in an unbounded liquid water phantom, the source was immersed at the center of a cube phantom of 100 cm^3^. The liquid water density was 0.998 g/cm^3^, and photon histories of up to 10^10^ were used to obtain the results with a standard deviation of less than 0.5% (k = 1). The obtained dose rate constant for the BEBIG ^60^Co source was 1.108±0.001 cGyh^-1^U^-1^, which is consistent with the values in the literature. The radial dose functions were compared with the values of the consensus data set in the literature, and they are consistent with the published data for this energy range.

## Introduction

The use of high-dose-rate (HDR) brachytherapy is a notably popular and acceptable practice around the world. The most common isotope source is ^192^Ir, but ^60^Co is also available for HDR. In a recently published study [1], the authors have compared the physical properties of ^60^Co and ^192^Ir HDR sources. They demonstrated that integral dose fall-off is higher for ^192^Ir than ^60^Co within the first 22 cm from the source. At larger distances the relationship is reversed. Their study suggests that no advantage or disadvantage exists for ^60^Co sources compared with ^192^Ir sources regarded to clinical aspects. Some of the main advantages of ^60^Co compared to ^192^Ir are the economic and practical benefits, which include its longer half-live of 5.27 years. With this longer half-live, significant cost savings may be achieved with ^60^Co, where source replacements are required every 3–4 years, whereas ^192^Ir requires 3–4 months. The equipment down-time and physics support time are also reduced by approximately 40% with ^60^Co compared to ^192^Ir [2].

Recently, Eckert & Ziegler BEBIG, Germany, introduced a new dual-source afterloading brachytherapy machine, which allows one to choose either a ^60^Co or a ^192^Ir source. Although ^60^Co was introduced decades ago, dosimetry data remain scarce [3]. The higher energy of the ^60^Co sources as an option to treat gynecological tumors has not been fully investigated.

It is well known that a dose rate table for a specific source model in water forms the basic dataset for clinical brachytherapy dosimetry. Then, these data can be used as the input for the treatment-planning system (TPS) and always as the gold standard to fully verify the TPS calculations. A specific dosimetric dataset should be used for a specific source model. The TG43 formalism and its update the TG43U1 [4,5] was initially designed for small low-dose-rate (LDR) interstitial sources, but it has been extended to high-dose-rate (HDR), pulsed-dose-rate (PDR) and other LDR sources. A dose rate table and the TG43 parameters and functions [6] have been widely accepted by researchers in the investigation and application of brachytherapy sources.

The dosimetry data are generated by Monte Carlo methods [4,5]. Selvam and Bhola [7] demonstrated that the dose-rate data compare well for distances larger than 0.5 cm but they have shown differences in dose values up to 9% for regions close to BEBIG ^60^Co source when compare with other works [8,9] for these sources.

In the AAPM and ESTRO report [10], it is indicated that the simulation of Granero *et al*. [7] shows some underestimation of the dose rate nearby the transverse axis of the source and that Selvam and Bhola [8] are not symmetric at large distances from source. Therefore, consensus data recommendations were: the average values quoted in these works for the dose rate constant, the values provided by Selvam and Bhola for the radial dose function and those for Granero *et al*. for 2D anisotropy function. It is worth point out that for radial dose function the values chosen were actually those provided by Selvam and Bhola [7] for r ≤ 1 cm and by Granero et al. for r ≥ 1 cm [11].

The purpose of the present study is to use the Electron Gamma Shower (*EGSnrc*) Monte Carlo code, which was designed by National Research Council of Canada, to calculate the dosimetric parameters of the BEBIG ^60^Co source. This code is widely accepted as a computational tool for radiotherapy dose calculations because it has been thoroughly tested. In this work, *EGSnrc* is used to calculate the dose rate distributions around the BEBIG ^60^Co HDR source in an unbounded liquid water phantom, and the results are compared to recent published data [5].

## Materials and Methods

### EGSnrc code system

The Monte Carlo code used in this work was *EGSnrc* (Electron Gamma Shower) [12]. It is a general purpose Monte Carlo code system used for the simulation of the coupled transport of electrons and photons through an arbitrary geometry for particle energies ranging from 1 keV to 10 GeV. It is an improved version of its predecessor EGS4 [13] system with significant advances in several aspects of electron transport: new electron transport algorithm PRESTA-II, improved multiple-scattering theory which includes relativistic spin effects in the cross section, electron impact ionization, more accurate boundary crossing algorithm, and improved sampling algorithm for a variety of energy and angular distributions. In particular, it incorporates significant improvements in the implementation of condensed history technique for the simulation of charged particle transport and better low energy cross sections. The code contains a multi-platform version [14] with several user codes like: DOSRZnrc which scores dose in a generalized cylindrical geometry and FLURZnrc which scores particle fluence in the same geometry.

In April 2005, a geometry package to implement almost arbitrary geometries was added to the EGSnrc code system: egspp [15]. It comprises a C++ geometry library for defining the geometry of complex simulation environments and particle sources.

The EGSnrc C++ class library egspp provides: a general purpose geometry package that can be used to model a wide range of geometrical structures, a set of particle sources that can be used to simulate all sources available with the RZ series of user codes and DOSXYZnrc, a set of basic scoring classes, base application classes for developing simple and advanced applications. By deriving from these classes it is much easier to create a new C++ user code for EGSnrc.

The EGSnrc C++ has four user codes: cavity, egs_chamber, egs_fac and egs_cbct. In this work we used the code cavity to proceed the calculations and we used the C++ geometry package to simulated the source and the cubic phantom.

### Geometry Design of the ^60^Co BEBIG HDR source

**Fig 1:**
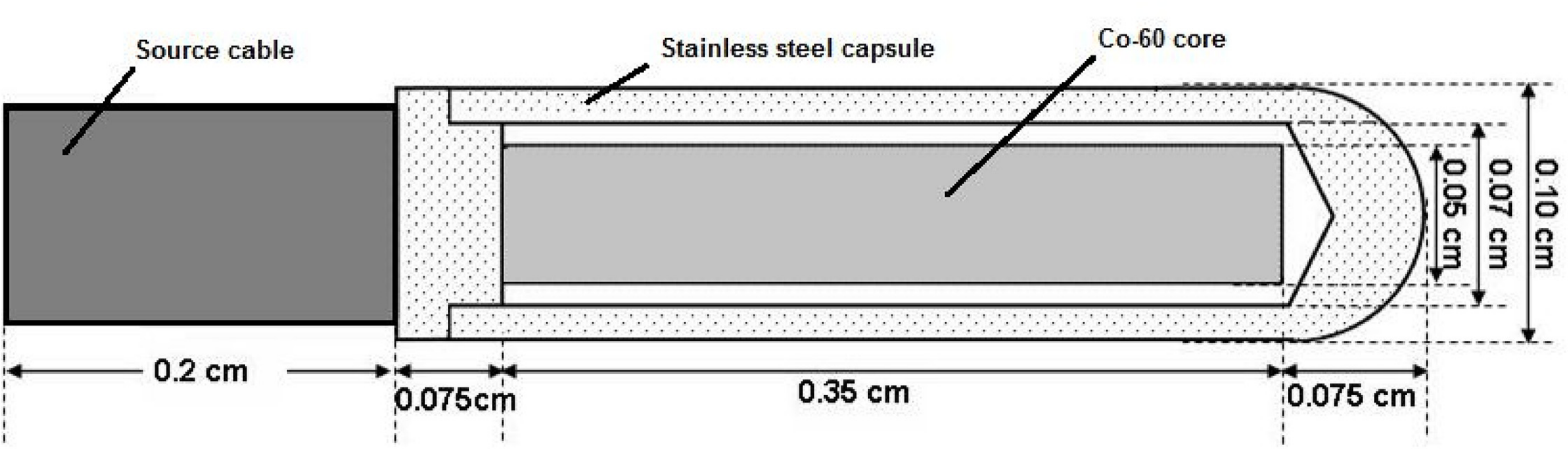
Geometry design of the ^60^Co source model Co0.A86. The coordinate axes in this study are also shown with their origin at the geometric center of the active volume. The dimensions are in millimeters [8].

The geometric design and material details of the BEBIG ^60^Co source, which was provided by the manufacturer, were used to construct the Monte Carlo geometry source. The source in this work for the Monte Carlo calculations was the new ^60^Co source (model Co0.A86), which is referred to as the new BEBIG ^60^Co HDR source and is a modified version of the ^60^Co source (model GK60M21). The geometry design is shown in Fig 1.

**Fig 2:**
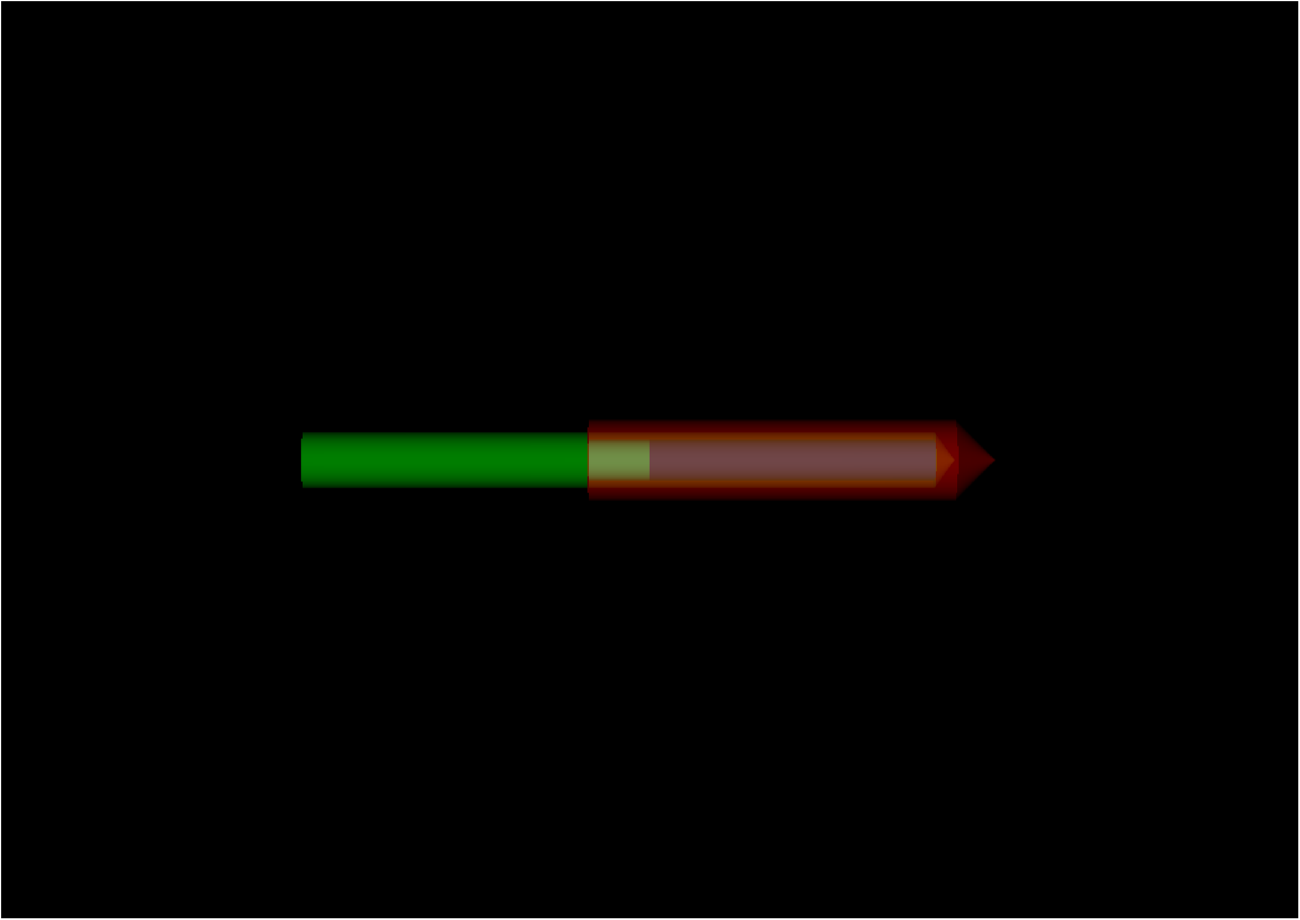
Simulation geometry of the BEBIG ^60^Co source. The figure was obtained using the egs-_view in the egs++ code.

The source is composed of a central cylindrical active core, which is made of metallic ^60^Co, 3.5 mm long and 0.5 mm in diameter. The active core is covered by a 0.15 mm thick cylindrical stainless-steel capsule with an external diameter of 1 mm [8], and its simulated geometry on *EGSnrc* is shown in Fig 2.

### Monte Carlo Calculations

To obtain the air-kerma and absorbed dose for the ^60^Co source, the *EGSnrc* spectrum, which was used to simulate ^60^Co, was composed of two photon energies: 1.17 and 1.33 MeV. Only the gamma part of the spectrum was used; the contribution of the electrons to the dose is negligible because of the full absorption by the stainless-steel wall around the metallic ^60^Co, where the electrons are stopped [8]. ^60^Co radionuclide was considered to be uniformly distributed in the active core of length L = 3.5 mm. To obtain the dose and kerma, one code in the *EGSnrc* package was used: *cavity*. Two score options were used: *HVL* and dose. The *HVL* scoring option and the dose were used to calculate the kerma and the absorbed dose around the source, respectively.

The *cavity* code calculates the kerma using the linear track-length kerma estimator, which speeds up the calculations and reduces statistic uncertainties. For this estimation, the ^60^Co source was located in the center of a 2.5x2.5x2.5 m^3^ air cube. The mass absorption coefficients for air in this calculation were calculated based on the recommended composition of TG-43U1 (40% humidity) [4,5]. The air-kerma was scored at 100 cm on the transverse axis in a cylindrical ring cell, which was 2 cm thick and 1 cm high. In addition, 10^9^ photon histories were required to obtain an uncertainty below 0.5%.

To obtain the dose distributions and the simulation geometry, the ^60^Co source was located at the center of a cubic phantom of 100 cc, which acts as an unbounded phantom. The liquid water density was 0.998 g/cm^3^ as recommended in the TG-43 U1 report [4,5]. The code that was used was *cavity* with the option “dose”. The dose was estimated on the transverse axis at sixteen positions: 0.25, 0.5, 0.75, 1, 1.5, 2, 3, 4, 5, 6, 7, 8, 10, 12, 15 and 20 cm. The score size voxel was 0.1 mm. To calculate the radial dose function and the dose rate constant of the source, 10^10^ histories were required to obtain an uncertainty below 0.5%.

The XCOM photon cross-section library was used in subsequent simulations. Consequently, the photoelectric effect, pair production, Rayleigh scattering and bound Compton scattering were included in the simulation. Variance reduction techniques were also used in the simulations to speed up the calculation. The cut-off energy was 10 keV for electrons and photons.

### Dose calculation formalism

The AAPM Task Group 43 [4] and its update [5] comprise currently accepted protocol for calculation of dose to water D_w_ in brachytherapy. The protocol provides a formalism to convert S_k_ to D_w_ at the point of interest using several calculated or measured factors.

Some important definitions are used in the protocol such as: the transverse plane of cylindrically symmetric source is that plane which is perpendicular to the longitudinal axis of the source and bisects the radioactivity distribution. A line source is a dosimetric approximation whereby radioactivity is assumed to be uniformly distributed along 1D line segment with active length L. While not accurately characterizing the radioactivity distribution within an actual source, this approximation is useful in characterizing the influence of inverse square law on a source’s dose distribution for the purposes of interpolating between or extrapolating beyond tabulated TG-43 [4,5] parameters values within clinical brachytherapy treatment planning systems.

The TG-43 [4,5] formalism establishes that the absorbed dose rate in a medium at a distance r from the source center and at an angle θ relative to the longitudinal axis should be expressed as:

$$\overset{\cdot }{D}(r,\theta )=S_{K}.\Lambda .\frac{G_{L}(r,\theta )}{G_{L}(r_{0},\theta_{0})}.g_{L}(r).F(r,\theta )$$ (1)

where S_K_ is the source air-kerma strength, Λ is the dose constant, G(r,θ) is the geometry factor that accounts for the distribution of the radioactive material, F(r,θ) is the anisotropy function that accounts for the angular dependence of photon absorption and scattering, and g(r) is the radial dose function that accounts for radial dependence of photon absorption and scattering in the medium along the transverse axis (θ = 90°). The reference point (r0, θ_0_) is r = 1 cm and θ_0_ = 90°. For the source studied here, the geometry factor can be approximated by:

$$G_{L}(r,\theta )=\frac{\beta }{Lr\;\text{sin}\;\theta }=\frac{\beta_{1}-\beta_{2}}{Lr\;\text{sin}\;\theta }$$ (2)

where L is the active length of the source and β is the angle subtended by the active source with respect to the point (r,θ). The subscript “L” in equation has been added to denote the line source approximation used for the geometry function.

After S_K_ and D(r,θ) are calculated, Λ, g_L_(r), and F(r,θ) can be formulated as follows:

$$\Lambda =\frac{\overset{\cdot }{D}(r_{0},\theta_{0})}{S_{K}}$$ (3)

$$g_{L}(r)=\frac{\overset{\cdot }{D}(r,\theta_{0})}{\overset{\cdot }{D}(r_{0},\theta_{0})}\frac{G_{L}(r_{0},\theta_{0})}{G_{L}(r,\theta_{0})}$$ (4)

$$F(r,\theta )=\frac{\overset{\cdot }{D}(r,\theta )}{\overset{\cdot }{D}(r,\theta_{0})}\frac{G_{L}(r,\theta_{0})}{G_{L}(r,\theta )}$$ (5)

The radial dose function, g_L_(r) accounts for dose fall-off on the transverse-plane due to photon scattering and attenuation in water medium. The function is also influenced by the geometry factor, G_L_(r,θ) and the anisotropy factor F(r, θ). The geometry factor depends on the physical parameters of the source, i.g, the length and the radius of the source. An identical construction of the sources can ensure same geometry factors. The isodose curve is influenced by anisotropy factor in clinical dose distribution. The anisotropy function describes the variation in dose as a function of polar angle relative to transverse plane.These two functions are essential for comparing different brachytherapy sources.

### Dose Rate Constant and Radial Dose Function Calculations

The dose rate constant Λ was calculated by dividing the dose-to-water per history in a 0.1-mm voxel, which was centered at the reference position of 1 cm and 90° in the 100x100x100 cm^3^ water phantom, by the air times d^2^ per history. Similar to the studies of Medich *et al*. [16,17], the air kerma per history in this study was calculated in air on the transversal axis at 100 cm from the source, as previously described. This technique differs from the extrapolation technique that is used in most studies of ^192^Ir sources [18], where the air kerma strength is scored in air along the transverse axis, fitted to a linear function and subsequently extrapolated to zero distance to correct for scatter and attenuation in air. Following this theory, the air kerma strength was calculated using Eq 6.

$$S_{K}=\overset{\cdot }{K_{air}}(100cm).d^{2}$$ (6)

where *S*_*k*_ is the air kerma strength of the BEBIG ^60^Co source, *K*_*air*_ is the air kerma at 100 cm on the transversal axis of the source, and *d* is the distance to source, which is 100 cm. The air kerma strength was expressed in units of 1U = 1 μGyh^-1^m^2^.

Then, the dose rate constant was obtained using Eq 3.

The radial dose function *g*_*L*_ was calculated using a line source geometry function at sixteen distances over a range of 0.2–20 cm. To obtain the radial dose function, the geometric factor *G*_*L*_ *(r*,*θ)* was used with a length *L* of 3.5 mm as described in Eq 2. The radial dose function was calculated using Eq 4. The radial dose function quantifies the correction for attenuation in water as a function of the radial distance after the dependence can be corrected by the inverse square law of the distance, which is calculated with the geometry function of the source.

## Results

The uncertainties in the final dose rate distributions for ^60^Co sources can be decreased to only the statistical uncertainties (type A). Thus, in this study, the final uncertainty with k = 1 is less than 1% for all points except for the points that are notably far from the source, whose final uncertainty is 2.5%. Type B uncertainties were not included in the present work. A detailed discussion about their estimation for brachytherapy source simulation as well as the difficulties inherent to their evaluation can be found in TG-43U1 [5] and TG-138 [19].

The obtained radial dose function and dose rate constant of the BEBIG ^60^Co source in this work were compared with those obtained by Granero et al.(S1 File)[5] and Selvam and Bhola (consensus data)[7], as shown in Table 1. Granero *et al*. obtained the results by using the GEANT4 code and Selvam and Bhola were obtained by simulations using DOSRZ a code that is part of *EGSnrc*.

**Table 1:** Dose Rate Constant of the BEBIG ^60^Co source.

	Λ (cGyh^-1^U^-1^)
**This work**	1.108±0.001
Granero *et al*.	1.1087±0.0011
Selvam and Bhola	1.097

Table 1 shows a consistency better than 1% between the published values of Granero *et al*. and the calculated values in this work. With the values of Selvam and Bhola show a consistency of an 1% with the calculated values in this work.

**Fig 3:**
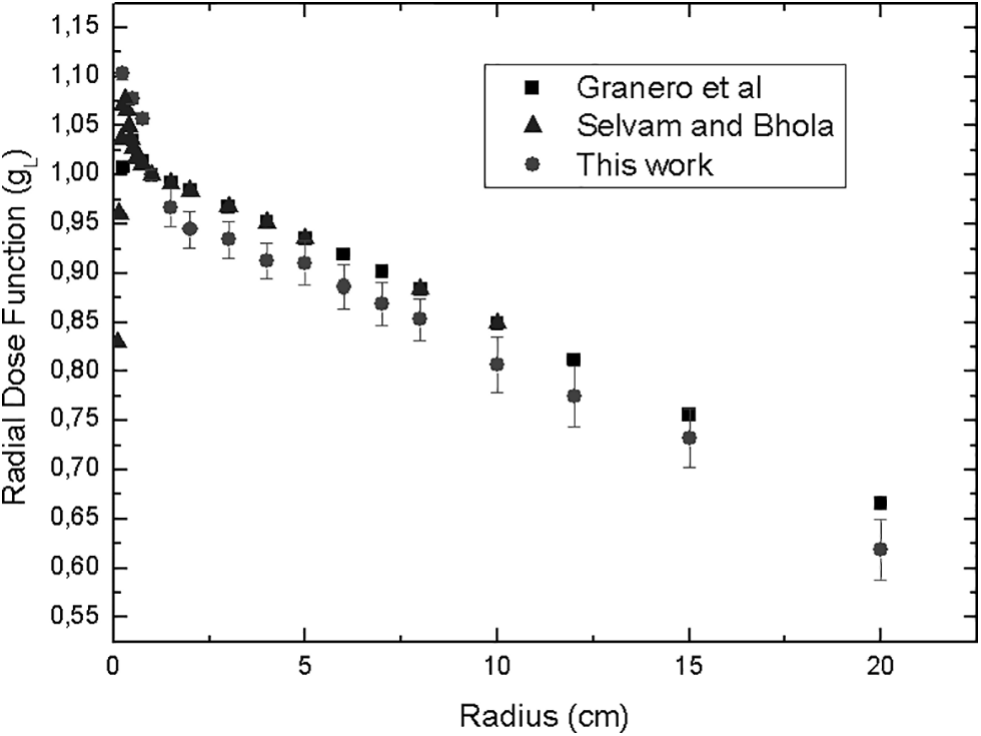
Comparison of the radial dose function in the source transversal axis, which was obtained for the BEBIG ^60^Co HDR source using the *cavity EGSnrc* Monte Carlo code.

The graph of the radial dose function of this work is shown in Fig 3 with the values obtained by Granero *et al*. and Selvam and Bhola. The points in the graph show increasing uncertainties when the radius distance increases and the number of histories remains constant. The maximum uncertainty of 3% was found for a radius distance of 20 cm, and a minimum uncertainty of 0.5% was found for a radius distance of 0.25 cm.

## Conclusions

In this study, the dose rate constant and radial dose function were calculated for the BEBIG ^60^Co source in an unbounded liquid water phantom using the *cavity* code. The dose rate constant is consistent with the results of Granero *et al*. and Selvam and Bhola within 1%. Dose rate data are compared to GEANT4 and DORZnrc Monte Carlo code. However, the radial dose function is different by up to 7% for the points that are notably near the source on the transversal axis because of the high-energy photons from ^60^Co, which causes an electronic disequilibrium at the interface between the source capsule and the liquid water for distances up to 1 cm. In their work, Selvam and Bhola analyzed how the cable length considered in the geometry could affect the dose rates. They argued that Granero *et al*. had considered a cable of 1 mm instead of 5 mm cable they indicated and this could explain the disagreement between the two calculations in r< 1.0 cm. This work considered a size cable of 5 mm. The results indicate that the cable length is not the only parameter that could lead to these discrepancies.

The radial dose function is different by up to 9% for the point 0.25 cm, 4.5% for 0.5 cm and 4% for 0.75 cm near the source if we consider the result of Granero *et al* but if we consider the Selvam and Bhola this discrepancies diminishes to 2.5%, 3.5 and 3% for 0.25, 0.5 and 0.75 cm point near the source. Today the data consensus is the results of Selvam and Bhola up to 1 cm near the source and the results of Granero *et al*. to 1cm up to 20 cm. This difference about 6% between the Selvam and Bhola result and Granero is assigned to the cable length. Even so our result is different in average about 4% if we consider all points of the radial dose function. This difference could be assigned to a differences and difficulties to handle with the cavity user code that is in the package of EGSnrc. The work uses a change in the cavity code to enable to calculate the absorbed dose.

The results of Selvam and Bhola were obtained with EGSnrc also but with another type of user code that is DOSRZ. This code is used to obtain dose and kerma in a cylindrical geometry and the details of the source could not be achieved as in the egs++. In fact, the difference about the results could not be explained about this. For regions where electronic equilibrium exists the results show a good agreement for most calculation points with the published results.

The cut off energy used for this work was 0.521 MeV to electrons and 10 keV to photons and we consider the dose to obtain the radial dose function. For the souce depending on the radius the statistical uncertainty vary between 0.1% to 2% for the points located close to the source and the other far from the source.

The other details such as Monte Carlo code could affect the final results. This work used an *EGSnrc* with a code *cavity* to obtain these dose rates; the source geometry was a line source approximation. This code could underestimate the dose nearby the source. For the calculation at greater distances, a difference of 7% was observed at the 20 cm radius most likely because the dosimetric data were obtained under full-scatter conditions, which affects the dose value at distances greater than 5 cm from the source. In general, the comparison shows good consistency with the results of Granero *et al*., and the egs++ Monte Carlo code in conjunction with the *cavity code* can be used to estimate these factors.

## Supporting Information

S1 File Granero Data. (JPG)
