# Supplementary figures and images for: Monte Carlo Dosimetry of the 60Co BEBIG High Dose Rate for Brachytherapy
Source: PLoS One. 2015 Sep 29;10(9):e0139032. doi: 10.1371/journal.pone.0139032 (PMC4587958; doi:10.1371/journal.pone.0139032)

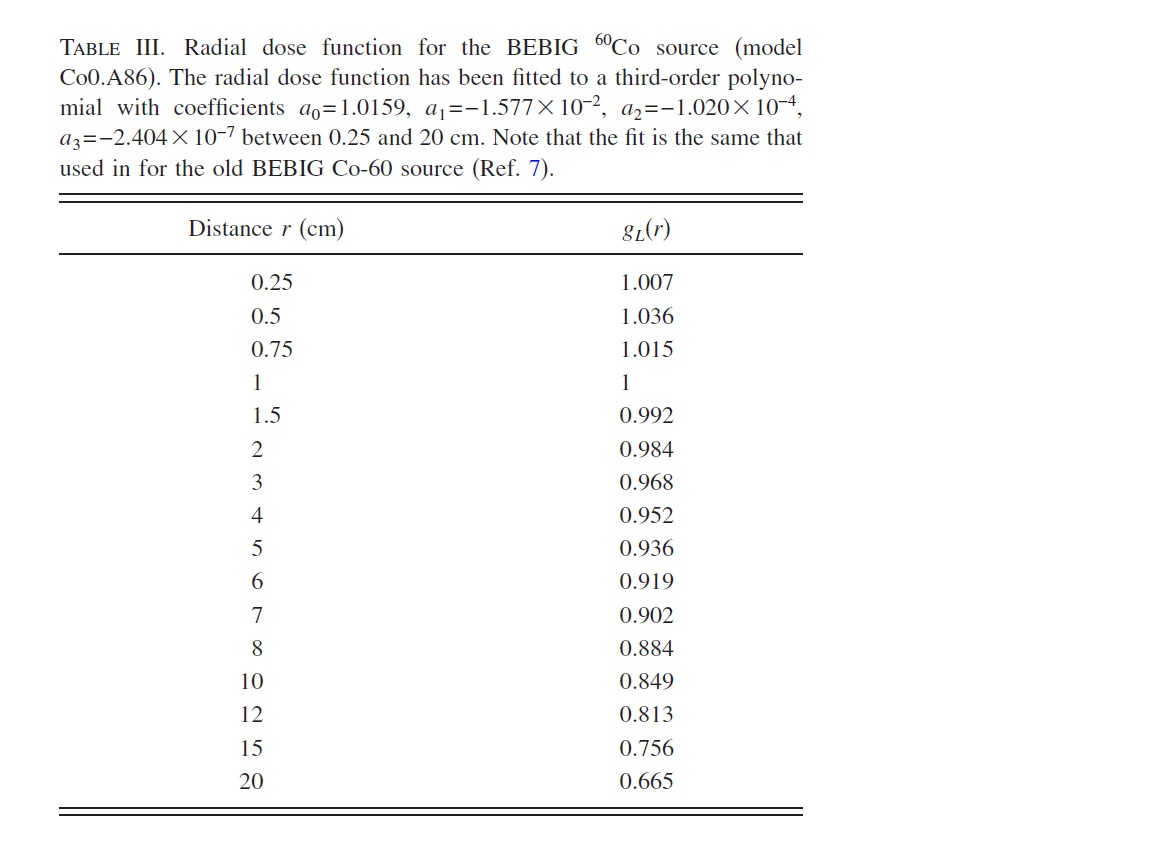

Supplement: S1 File — (JPG) [file pone.0139032.s001.jpg]
